# Anti-Inflammatory Activity Is a Possible Mechanism by Which the Polyherbal Formulation Comprised of *Nigella sativa* (Seeds), *Hemidesmus indicus* (Root), and *Smilax glabra* (Rhizome) Mediates Its Antihepatocarcinogenic Effects

**DOI:** 10.1155/2012/108626

**Published:** 2012-11-13

**Authors:** Prasanna B. Galhena, Sameera R. Samarakoon, M. Ira Thabrew, G. A. K. Weerasinghe, Mayuri G. Thammitiyagodage, W. D. Ratnasooriya, Kamani H. Tennekoon

**Affiliations:** ^1^Department of Biochemistry and Clinical Chemistry, Faculty of Medicine, University of Kelaniya, Ragama, Sri Lanka; ^2^Institute of Biochemistry, Molecular Biology, and Biotechnology, University of Colombo, Colombo 03, Sri Lanka; ^3^Department of Physiology, South Asian Institute of Technology and Medicine, Malabe, Sri Lanka; ^4^Animal Research Centre, Medical Research Institute, Colombo 08, Sri Lanka; ^5^Department of Zoology, Faculty of Science, University of Colombo, Colombo 03, Sri Lanka

## Abstract

The present study investigated the anti-inflammatory effects of a polyherbal decoction comprised of *Nigella sativa, Hemidesmus indicus*, and *Smilax glabra* in order to justify its claimed antihepatocarcinogenic activity. Activation of hepatic nuclear factor-kappa B (NF-**κ**B), I**κ**B kinase (IKK **α**/**β**) proteins, and TNF**α** and IL-6 expression was investigated in diethylnitrosamine- (DEN-) induced C3H mice-bearing early hepatocarcinogenic changes. Acute phase inflammatory response was evaluated by carrageenan-induced rat paw edema formation. Anti-inflammatory mechanisms were also assessed by determining effect on (a) membrane stabilization, (b) nitric oxide (NO) inhibitory activity, and (c) inhibition of leukocyte migration. A significant inhibition of the paw edema formation was observed in healthy rats as well as in rats bearing early hepatocarcinogenic changes with oral administration of the decoction. As with the positive control, indomethacin (10 mg/kg b.w.) the inhibitory effect was pronounced at 3rd and 4th h after carrageenan injection. A notable IKK **α**/**β** mediated hepatic NF-**κ**B inactivation was associated with a significant hepatic TNF**α** downregulation among mice-bearing hepatocarcinogenic changes subjected to decoction treatment. Inhibition of NO production, leukocyte migration, and membrane stabilization are possible mechanisms by which anti-inflammatory effect is mediated by the decoction. Overall findings imply that anti-inflammatory activity could be one of the mechanisms by which the decoction mediates its antihepatocarcinogenic effects.

## 1. Introduction

Prolonged inflammation is thought to set up a cycle of liver cell destruction and regeneration, resulting in a mitogenic and mutagenic environment that precipitates random genetic and chromosomal damage and leads to the development of HCC [1, 2]. Recently, continuous intrahepatic inflammation has been reported to be the principal oncogenic factor in hepatocarcinogenesis [3, 4]. It has also been confirmed that the tumor microenvironment, which is largely orchestrated by inflammatory cells, is an indispensable participant in the neoplastic process, fostering proliferation, cell survival, and migration [5, 6]. Nuclear factor kappa B (NF-*κ*B) is a vital transcriptional factor which links inflammation and HCC. It has previously been reported that the inhibition of hepatic NF-*κ*B activity could significantly reduce HCC development in Mdr2^−/−^ mice [7]. Further, the activation of NF-*κ*B upregulates the expression of an array of downstream targets that are involved in tumor promotion, angiogenesis, and metastasis [8, 9]. Therefore, these insights support the need for anti-inflammatory therapeutic approaches to the development of new anticancer drugs. 

During the past few years, many naturally occurring anti-inflammatory or immunomodulatory plant metabolites have been reported to mediate anticancer effects by inducing or suppressing specific cellular inflammatory activities and the associated molecular signaling pathways [10]. In many Asian countries including Sri Lanka, polyherbal preparations are also used traditionally to treat cancer patients despite the lack of evidence from scientifically controlled studies to prove or disprove these claims. One such remedy, used by a particular family of ayurvedic physicians in Sri Lanka, is a decoction comprised of *Nigella sativa *(seeds)*, Hemidesmus indicus* (root), and *Smilax glabra* (rhizome). The seed of *N. sativa *has been used as a folk medicine for the treatment of a number of illnesses and conditions that include bronchial asthma, cough, rheumatism, hypertension, diabetes, inflammation, eczema, fever, tumor, and influenza [11, 12]. *Hemidesmus indicus *(root) is an ingredient in ayurvedic preparations against blood diseases and inflammation [13]. Rhizome of *Smilax glabra is *commonly used clinically to prevent leptospirosis and to treat syphilis, acute bacterial dysentery, acute and chronic nephritis, mercury poisoning, and rheumatoid arthritis [14]. Recent *in vivo* investigations in rats have confirmed that the above decoction can significantly protect rat livers against carcinogenic changes mediated by diethylnitrosamine (DEN) without producing any toxic side effects [15]. It has also been reported that this decoction can induce significant cytotoxicity against human hepatoma HepG2 cells *in vitro *[16, 17]. However, the exact mechanisms by which the above decoction mediates its anticarcinogenic action have not completely been demonstrated to date.

In the present study, the anti-inflammatory effect of the decoction comprised of *N. sativa* seeds, *H. indicus* roots, and *S. glabra* rhizome was evaluated in order to understand the mechanisms by which it mediates the claimed antihepatocarcinogenic effect. To achieve this objective, the ability of the decoction to modulate host immune response towards diethylnitrosamine-(DEN-) induced early hepatocarcinogenesis was investigated by evaluating its effect on (a) carrageenan-induced rat paw formation, (b) NO production by rat peritoneal cells, (c) leukocyte migration during carrageenan-induced rat peritonitis, (d) expression of intrahepatic inflammatory mediators (TNF*α* and IL-6), and (e) key inflammatory regulator; nuclear factor-kappa B (NF-*κ*B) activation.

## 2. Methods and Materials

### 2.1. Chemicals

Diethylnitrosamine (DEN), trypan blue, pentobarbital sodium USP, carrageenan, sodium citrate, sodium chloride, citric acid, sulfanilamide, N-1-naphthylenediamine dihyrochloride, phosphoric acid, and N-monomethyl-L-arginine acetate (NMMA) were purchased from Sigma Aldrich (St. Louis, MO, USA). RPMI 1640 medium was obtained from GIBCO, Life Technologies (Paisley, Scotland). All other chemicals and reagents were of analytical grade.

### 2.2. Experimental Animals

Healthy male wistar rats (six-week-old littermates, 190 ± 10 g), and healthy male C3H mice (six-week-old littermates, 25 ± 5 g) were obtained from the Medical Research Institute, Colombo, Sri Lanka. Animals were housed in polypropylene cages (four rats per cage) and maintained at 25 ± 2°C with 12 h dark cycle. They were fed *ad libitum *with standard laboratory diet, prepared by the Medical Research Institute, based on a formula recommended by the W H O and water [18, 19]. All animal experiments were ethically approved by Ethics Review Committee of Medical Research Institute, Sri Lanka and were conducted in accordance with Ethical Guidelines for studies involving animals in Sri Lanka [19]. 

### 2.3. Plant Materials

Dried seeds of *N. sativa* (family Ranunculacaea), dried rhizome of *S. glabra* (family Smilacaceae), and dried roots of *H. indicus* (family Asclepiadaceae) were purchased from D. J. Fenando Pvt Ltd., Gabose lane, Colombo and authenticated by the botanist at the Bandaranayake Memorial Ayurvedic Research Institute (BMARI), Nawinna, Maharagama, Sri Lanka. Voucher specimens (UKFM/B/2006/01, UKFM/B/2006/02, UKFM/B/2006/03) have been deposited at the Department of Biochemistry and Clinical Chemistry, Faculty of Medicine, University of Kelaniya, Sri Lanka.

### 2.4. Preparation of Standardized Decoction

Preparation of the standardized decoction was carried out according to the method previously described by Samarakoon et al. [16]. Briefly, equal portions (20 g each) of *N. sativa *(seeds), *H. indicus *(roots), and *S. glabra *(rhizome) were mixed and boiled gently in 1.6 l of distilled water until a final volume of 200 mL was obtained. The extract was then filtered, freeze dried, and stored in a vacuum desiccator at −4°C. The percentage yield of the freeze dried plant material was approximately 15.242 ± 0.319%. 

### 2.5. Dosage and Administration of Decoction to Animal Models

The decoction at a dose of 1 g/kg body weight (b.w)/day and 1.5 g/kg b.w./day was administered orally to rats and mice, respectively, via sondi needle. These doses corresponded to the normal therapeutic dose administered to adult human as calculated based on relative surface areas of human and each individual animal (rats and mouse) [20]. 

### 2.6. Investigation of Anti-Inflammatory Activities of the Decoction

Anti-inflammatory potential of the decoction was investigated by (a) *in vivo *carrageenan*-*induced rat paw edema model [21, 22] and carrageenan-induced rat peritonitis model, (b) *in vitro *human red-cell membrane stabilization [23] and NO production by rat peritoneal cell cultures.

#### 2.6.1. Carrageenan-Induced Rat Paw Edema

Effects of oral administration of the decoction on carrageenan-induced rat paw edema were assessed in healthy rats as well as in rats with chemically induced early hepatocarcinogenic changes.

**Experimental Design**

Fifty wistar rats (*n* = 50) were selected and randomly divided into four groups (three groups of ten rats each and a fourth group of twenty rats). Groups 1, 2, and 3 served as the normal control, decoction control, and positive control (the group treated with indomethacin at a dose of 10 mg/kg b.w.), respectively, while Group 4 was subjected to initiation of hepatocarcinogenic changes according to liver medium term bioassay protocol, previously described [24]. Briefly, on day 1, each animal in Group 4 was injected with a single intraperitoneal DEN (200 mg/kg body weight) injection and then divided into two subgroups (4a; DEN control, 4b; Test) consisting of ten rats (*n* = 10) in each group. Twenty-four hours after DEN injection, each animal of group 4a was orally administered 1 mL of distilled water while group 4b received 1 mL of decoction at oral dose of 1 g/kg body weight via a sondi needle. At the same time, Group 1 was administered 1 mL distilled water per rat orally, while each animal of Group 2 received orally 1 mL of the decoction at a dose corresponding to 1 g/kg b.w.

One hour after decoction administration, edema was induced in the rat right hind paw by subcutaneous injection of 0.05 mL of 1% carrageenin in 0.9% saline. The volumes of the injected paws were measured 1 hr prior to the injection (*V*_0_) and 1, 2, 3, and 4 hrs (*V*_*t*_) after induction of inflammation using a Plethysmometer, (Model 7150, Ugo Basile). The percent inhibition of inflammation at each hour for each test group was calculated as follows:

(1) percent  (%)  inhibition=(Vt−V0)  in  control  rats−(Vt−V0)  in  treated  rats×100(Vt−V0)  in  control  rats.

#### 2.6.2. Effect of the Decoction on Human Red-Cell Membrane Stability

Membrane stability of red cells incubated with different decoction doses (500 *μ*g/mL, 250 *μ*g/mL, 125 *μ*g/mL, and 62.5 *μ*g/mL) was tested according to an *in vitro* model previously described by Trnavsky (1974) [25]. Briefly, fresh venous blood (1 mL) from a healthy volunteer was collected in a tube containing 1 mL of sterile Alsevier solution (2% Dextrose, 0.8% Sodium Citrate, 0.05% Citric acid, and 0.42% Sodium chloride). Separation of red-blood cells (RBC) was performed by centrifuging the blood samples at 3500 rpm for 10 min with three washes in freshly prepared sterile Alsevire solution. Finally, a 10% RBC working solution was prepared by adding 9 mL of sterile Alsevier solution to the pellet remaining in the tube.

A reaction mixture (4.5 mL) consisting of 2 mL hypotonic saline (0.25% w : v NaCl), 1 mL 0.15 M sodium phosphate buffer (pH 7.4), and varying concentration of the decoction in 1 mL of normal saline was added with 0.5 mL of 10% HRBC in normal saline. Two controls were performed: one with 1.0 mL of isotonic saline instead of extract (control_1_) and the other one with 1 mL of extract solution without red-blood cells (control_2_). The mixture was incubated at 56°C for 30 min. The tubes were cooled under running water for 20 min, the mixture was centrifuged, and the absorbance of the supernatants was read at 560 nm. Diclofenac sodium (50 *μ*g/mL) was used as the reference drug. 

Percentage inhibition of the inflammation was expressed as

(2) $$\begin{array}{c} 100-\left(\frac{A_{560}\;\text{test}-A_{560}\;{\text{control}}_{2}\times 100}{A_{560}\;{\text{control}}_{1}}\right). \end{array}$$

#### 2.6.3. *In Vivo* Effect of the Decoction on Leukocyte Migration during Carrageenan-Induced Rat Peritonitis

A total of eighteen (*n* = 18) male wistar rats were equally divided into three experimental groups; distilled water control group (Group A), prednisolone positive control group (Group B), and decoction control group (Group C). Rats in groups A, B, and C were orally administered with distilled water, prednisolone at a dose of 5 mg/kg b.w., and decoction at a dose of 1 g/kg body weight, respectively. After 1 h, peritonitis was induced in rats by single intraperitoneal administration of 1% solution of carrageenan (dissolved in PBS; pH 7.4) at a dose of 5 mg/kg b.w. Two hours later, peritoneal cavity of each rat was washed with 20 mL of Ca^2+^ and Mg^2+^ free sterile saline and the content was drained by 18 G cannula. The peritoneal fluid was centrifuged at 150 g for 5 min, supernatant; was discarded and the cell pellet was resuspended in 1 mL of RPMI 1640 supplemented with 0.2% sodium bicarbonate, 10% FBS, streptomycin (100 units/mL), and penicillin (100 units/mL). The total leukocyte count was determined in the peritoneal fluid recovered from each rat in three experimental groups [26]. 

#### 2.6.4. *In Vitro* Effect of the Decoction Rat Peritoneal Cell Viability and NO Production

**(1) In Vitro Cell Viability Test**

Effect of the decoction on rat peritoneal cell viability was evaluated by trypan blue exclusion assay [27]. Briefly, rat peritoneal cells were plated in 96-well plate (0.2 × 10^6^) and incubated with different concentrations of the decoction (1600, 1200, 600, and 300 *μ*g/mL; *n* = 6 per each concentration) for 0, 1, and 24 h at 37°C in a 5% CO_2_ incubator. After each incubation time period, cell suspension was centrifuged at 150 g for 5 min, 180 *μ*L of the supernatant removed by aspiration, and 20 *μ*L of 0.2% trypan blue added and incubated at room temperature for 5 min. The number of trypan blue stained (dead) cells and unstained cells (live) was counted using a hemocytometer. As the control, peritoneal cells were incubated with RPMI 1640 supplemented with 10% FBS for each incubation period as mentioned above.

**(2) In Vitro NO Production**

Inhibitory effect of NO production by the decoction was performed according to the method previously described by Pereira Nacife et al. [28]. Briefly, rat peritoneal cells cultured in RPMI 1640 were seeded in 12-well tissue culture plate at a cell density of 1 × 10^6^ cells/mL and incubated at 37°C in CO_2_, incubator (5% CO_2_ ± 95% air) for 24 hrs. After 24 hrs, cells were treated with (a) decoction at doses of 300 *μ*g/mL, 600 *μ*g/mL, and 1200 *μ*g/mL (test) and (b) 1 mM N-monomethyl L-arginine acetate salt (NMMA) and nitric oxide synthase inhibitor (positive control). Cells were further incubated for 24 hrs at 37°C and harvested. Cell suspension was then centrifuged at 10,000 ×g for 10 min and the nitrite levels were assessed in the cells free clear supernatant.

**(3) Measurement of NO Production by Rat Peritoneal Cells**

NO production by rat peritoneal cells was determined by measuring nitrite (NO^2−^) in culture supernatant using the Griess reagent [28]. Briefly, 100 *μ*L from each cells free clear supernatant was transferred into 96-well microplates and mixed with equal volume of Griess reagent (1% sulfanilamide and 0.1% naphthalene diamine dihydrochloride in 1% orthophosphoric acid). After 10 min incubation, the plate was read on a ELISA reader (ELX 800, Bio-Tek Instruments Inc., Winooski, USA) at 540 nm. The concentrations of nitrite were derived from regression analysis using serial dilutions (100–0 *μ*M) of sodium nitrite as the standard.

### 2.7. Effect of the Decoction on Hepatic NF-*κ*B and I*κ*B Kinase Complex Activation

*In vivo *modulatory effect of the decoction on one of the key inflammatory regulators (NF-*κ*B) was investigated in healthy mice as well as in mice bearing DEN induced early hepato-carcinogenic changes. 

#### 2.7.1. Experimental Design

A total of thirty-two (*n* = 32) C3H mice (5-6 weeks old) were randomly divided into four equal groups.

**Figure 1:**
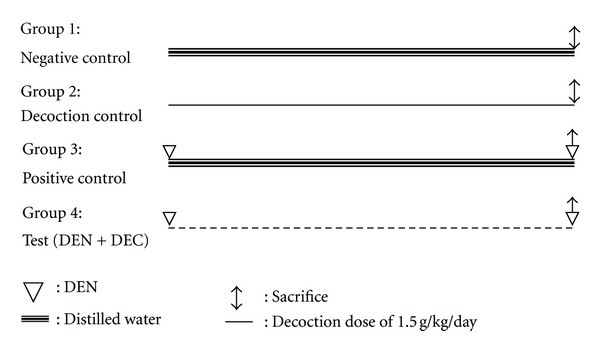
Schematic presentation of treatment schedule.

Mice in control group (Group 1) were orally fed with distilled water for a period of four weeks. Animals in decoction control group (Group 2) were orally administered with the decoction at a dose of 1.5 g/kg b.w./day for four weeks. Mice in the remaining two groups were injected with Diethylnitrosamine (DEN) at a dose of 20 mg/kg b.w. as a single intraperitoneal injection on day 1. From day 2 onwards, eight animals from DEN-induced group (test group) were orally fed with the decoction at a dose of 1.5 g/kg b.w./day, for a period of four weeks while the rest (positive control) were orally administered with distilled water for the same period (Figure 1). 

At the end of the fourth week, animals in test and positive control groups were subjected to a second dose of intraperitoneal DEN (20 mg/kg b.w.) half an hour before they were sacrificed in order to promote carcinogenic process. All animals in the four groups were anesthetized with a single intraperitoneal injection of pentobarbital at a dose of 40–85 mg/kg b.w. The liver sections from each mouse were obtained aseptically, and one portion was fixed with 10% formaldehyde and embedded in paraffin for immunohistochemistry studies while the rest was immediately frozen in liquid nitrogen and stored at −80°C for cytokine expression studies.

**(1) Immunohistochemical Analysis of NF-*κ*B and Ikk *α*/*β***

Each liver section was deparaffinized and hydrated by sequential immersion in xylene, graded alcohol solutions (100%, 95%, 80%, and 70%) for three minutes at each concentration, and running water for three minutes. Then sections were incubated in 3% hydrogen peroxide for 10 min to block the activity of endogenous peroxidases. Sections were washed with TRIS-HCl-buffered saline (TBS) before immersion in Antigen Retrieval Solution (10 mM sodium citrate buffer, pH 6 (ARS)) for 20 minutes in a water bath at 98°C. The slides with ARS solution were left at room temperature for 20 minutes before being washed with TBS three times for three minutes each. To reduce the background staining, the slides were immersed in biotin and avidin solution for 30 minutes. The sections were then further blocked with Bovine Serum Albumin (BSA) to reduce the nonspecific staining. Subsequently, slides were incubated with anti-p 65 rabbit polyclonal antibody for NF-*κ*B (sc-372) and anti-Ikk *α*/*β* rabbit polyclonal antibody for I kappa B kinase alpha/beta (sc-7607) supplied by Santa Cruz Biotechnology, Santa Cruz, CA, USA for 60 min at 37°C at an optimized dilution of 1 : 200. The sections were then washed three times with TBS for three minutes each before incubating for 60 minutes with goat antirabbit IgG-HRP (sc-3837, Santa Cruz Biotechnology, Santa Cruz, CA, USA) at an optimized dilution of 1 : 1000. The sections were washed again with TBS before finally incubating with DAB for 10 minutes. The sections were counterstained with Meyer's Hematoxylin and mounted with DPX for viewing under light microscope.

#### 2.7.2. Effect of the Decoction on mRNA Expression on Intrahepatic TNF*α* and IL-6 Selected Downstream Targets of NF-*κ*B

Total RNA from fifty milligrams of liver tissue was extracted according to guanidium isothiocyanate and acid-phenol extraction principle using TRIzol reagent (15596-018, Invitrogen Life Technologies, USA) [29]. Two micrograms of extracted liver RNA were reverse transcribed with 50 U of Moloney murine leukemia virus reverse transcriptase and then amplified using primers for murine TNF-*α*, IL-6, and glyceraldehydes 3-phosphate dehydrogenase (GAPDH) as an internal control. The sequence for each oligonucleotide primer was as follows: IL-6 forward primer 5′-CTG GTG ACA ACC GCC TTC CCT A-3′ and reverse primer 5′-ATG CTT AGG CAT AAC GCA CTA GGT-3′; TNF*α* forward primer 5′-GGC AGG TCT ACT TTG GAG TCA TTG C-3′ and reverse primer 5′-ACA TTC GAG GCT CCA GTG AAT TCG G-3′; GAPDH forward primer 5′-GAG GGG CCA TCC ACA GTC TTC-3′ and reverse primer 5′-CAT CAC CAT CTT CCA GGA GCG-3′. Optimized PCR conditions for each amplicon were 30 sec at 94°C, 30 sec at 56°C, 1 min at 72°C for 30 cycles (GAPDH amplicon), 45 sec at 94°C, 45 sec at 60°C, 1 min at 72°C for 35 cycles (TNF*α* amplicon), 1 min at 94°C, 1.5 min at 56°C, and 2 min at 72°C for 35 cycles (IL-6 amplicon). Amplified products were electrophoresed in 2% agarose gels and visualized after staining with ethidium bromide.

### 2.8. Statistical Analysis

The TNF*α* and IL-6 mRNA levels were normalized against the GAPDH mRNA level in the same sample. Results of RT-PCR were analyzed using Prism 2.01 (Graphpad Prism, San Diego, CA, USA). Mean ± SEM was used to describe data. Differences in mRNA expression in each group were determined by one-way ANOVA with Bonferroni. Comparison of all pairs of columns and the level of significance was set at *P* < 0.05. Results of anti-inflammatory experiments were described as mean ± SEM and differences were analyzed ANOVA followed by Bonferroni's test.

## 3. Results

### 3.1. Carrageenan-Induced Rat Paw Oedema

Results of the carrageenan induced rat paw oedema formation clearly indicated a strong anti-inflammatory effect mediated by the decoction (Table 1). Although an overall inhibition of rat paw oedema formation was observed with a single oral dose of the decoction (1 g/kg b.w.), the most significant effect was noted at 3rd h after carrageenan injection (32.1% inhibition; *P* < 0.01). However, the anti-inflammatory potency demonstrated by the decoction was less than that exhibited by indomethacin (10 mg/kg b.w.; 56.6% and 54.8% inhibition at 3rd and 4th h of postcarrageenan injection). This may be because indomethacin is a pure compound while the decoction is a crude extract containing several compounds. 

Single intraperitoneal DEN injection (200 mg/kg b.w.) also caused a significant inhibition of carrageenan induced rat paw edema. This inhibition was more potent than that of the indomethacin during 1st and 2nd h after carrageenan injection. However, oral administration of the decoction (1 g/kg b.w.) to rats bearing early hepatocarcinogenic changes induced by DEN showed a further inhibition of inflammation. This inhibition was more significant during late phase of inflammation (57.7% and 56.7% inhibition at 3rd and 4th hrs, resp.) and demonstrates that the decoction can exert anti-inflammatory activity even during early hepatocarcinogenic changes induced by DEN. 

### 3.2. Erythrocyte Membrane Stabilization

As observed in Table 2, decoction exhibited membrane stabilization effect in a dose-dependent manner (*r* ^2^ = 0.934) by inhibiting hypotonicity-induced lysis of erythrocyte membrane. A protective effect against erythrocyte membrane lysis is considered to be biochemical index of anti-inflammatory activity of any test compound [30]. The inhibitory effect of the decoction appears to be less potent than that of the reference drug, diclofenac sodium (73.9% inhibition at a dose of 50 *μ*g/mL). However, such a difference in the activity of the decoction may be due to presence of mixture of compounds in the decoction when compared with a single compound in diclofenac sodium. 

### 3.3. *In Vivo* Effect of the Decoction on Leukocyte Migration

**Figure 2:**
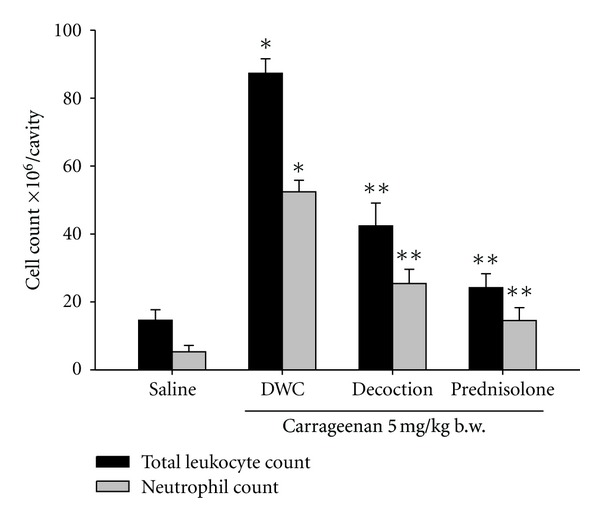
Inhibitory effect of the decoction on total leukocyte and neutrophil migration induced by carrageenan. Peritonitis was induced by single intraperitoneal injection of carrageenan and cells were counted 2 h later. Decoction (1 g/kg b.w.) was given orally 1 h before inflammatory stimuli. Values are given as mean ± S.E.M. (*n* = 6). *P < 0.05 compared to saline, **P < 0.05 compared to carrageenan (ANOVA followed by Bonferroni's post-test).

Single intraperitoneal injection of carrageenan (5 mg/kg b.w.) induced a significant leukocyte migration (86.27 ± 3.1 × 10^6^/cavity), when compared with the saline control group (6; 14.57 ± 1.8). However, a significant reduction in leukocyte migration was observed once rats were pretreated with the decoction at a dose of 1 g/kg b.w., thus confirming the presence of its claimed anti-inflammatory activity. The inhibition of leukocyte migration mediated by the decoction was not potent as that observed in rats pre-treated with prednisolne at a dose of 5 mg/kg b.w. (24.4 ± 4.1%) (Figure 2). 

### 3.4. *In Vitro* Cell Viability of Rat Peritoneal Cells

Viable cell count of rat peritoneal cells had reduced significantly, once incubated with highest decoction dose (1600 *μ*g/mL), thus indicating toxicity to rat peritoneal cells (Table 3).

Viability count for the rest of the decoction doses (1200 *μ*g/mL, 600 *μ*g/mL, and 300 *μ*g/mL) was within the range of 87%–94% and was comparable to viable cell count of control cells (>90% viability), indicating that these doses were well tolerated by rat peritoneal cells.

### 3.5. Inhibition of NO Production by Rat Peritoneal Cells *In Vitro*

**Figure 3:**
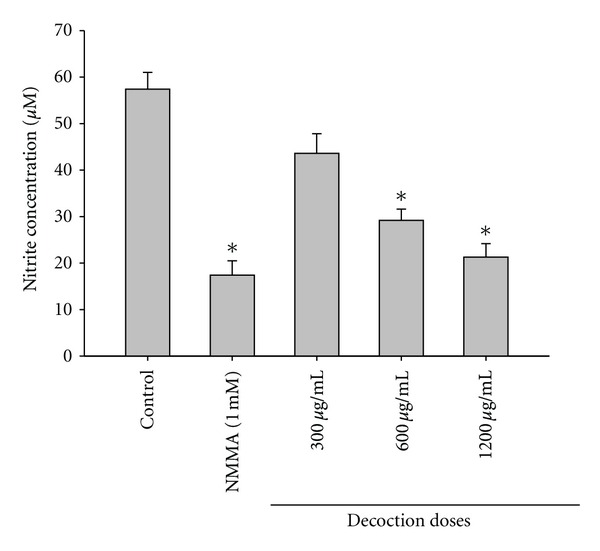
Effects of the decoction on *in vitro *NO production by rat peritoneal cells. Values are expressed as mean ± SEM, *n* = 6/concentration. *P < 0.05, when compared with control.

*In vitro *decoction treatment of peritoneal cells demonstrated a dose-dependent inhibition of NO production (*r* = 0.99, *P* < 0.05). Maximum inhibition by the decoction was observed at 1200 *μ*g/mL (21.3 ± 2.9%, *P* < 0.01). NMMA which is a nitric oxide synthase inhibitor also showed a comparable inhibition of NO production (17.4 ± 3.1%, *P* < 0.01) (Figure 3).

### 3.6. Effects of the Decoction on Activities of NF-*κ*B Transcription Factor and Ikk *α*/*β* Proteins in C3H Mouse Liver

The effect of the decoction on NF-*κ*B activation in C3H mice bearing early hepatocarcinogenic changes induced by DEN was assessed by immunohistochemical detection of the p 65 sub-cellular localization in hepatocytes according to method described in Section 2.7.1(1).

**Figure 4:**
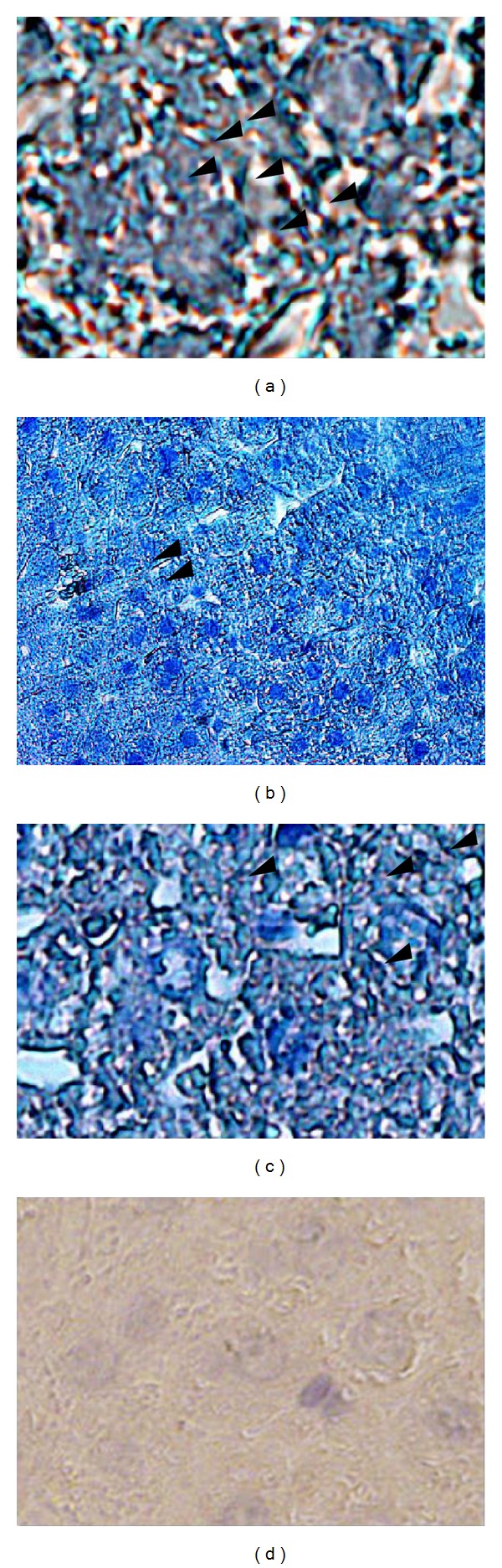
Immunohistochemical evaluation of NF-*κ*B nuclear translocation in hepatocytes of C3H mice injected with DEN and subsequently treated with the decoction. Liver sections were incubated with primary antibody to the p 65 subunit of NF-*κ*B as described in the methods. (a) After intraperitoneal injections of DEN (×100), (b) healthy mice (×40), (c) treated with the decoction after intraperitoneal injection of DEN (×100), and (d) negative control; section stained without primary antibody (×100).

As evident from Figure 4, predominant nuclear localization of p 65 protein was observed in hepatocytes of C3H mice in response to i.p. administration of DEN (positive control) in comparison with healthy mice. Oral administration of the decoction (1.5 g/kg b.w./day for 4 weeks), resulted in predominant cytoplasmic localization of p 65 protein in hepatocytes of DEN treated mice in comparison to mice injected with DEN only, thus indicating decoction mediated inhibition of NF-*κ*B nuclear translocation. A significant cytoplasmic localization of p 65 protein was also observed in hepatocytes of healthy mice. No reactive foci for p 65 protein were found in hepatocytes of C3H mice (untreated) incubated without primary p 65 anti-body (negative control). This confirms the absence of cross reactive foci in the tissue sections with secondary antibody. 

In order to determine whether the inhibitory effect of the decoction on NF-*κ*B was mediated via modulation of Ikk *α*/*β* proteins, activities of these proteins in mice injected with DEN and subsequently treated with the decoction (1.5 g/kg b.w./day) for four (4) weeks was examined in comparison to their activities in untreated mice injected with DEN. 

**Figure 5:**
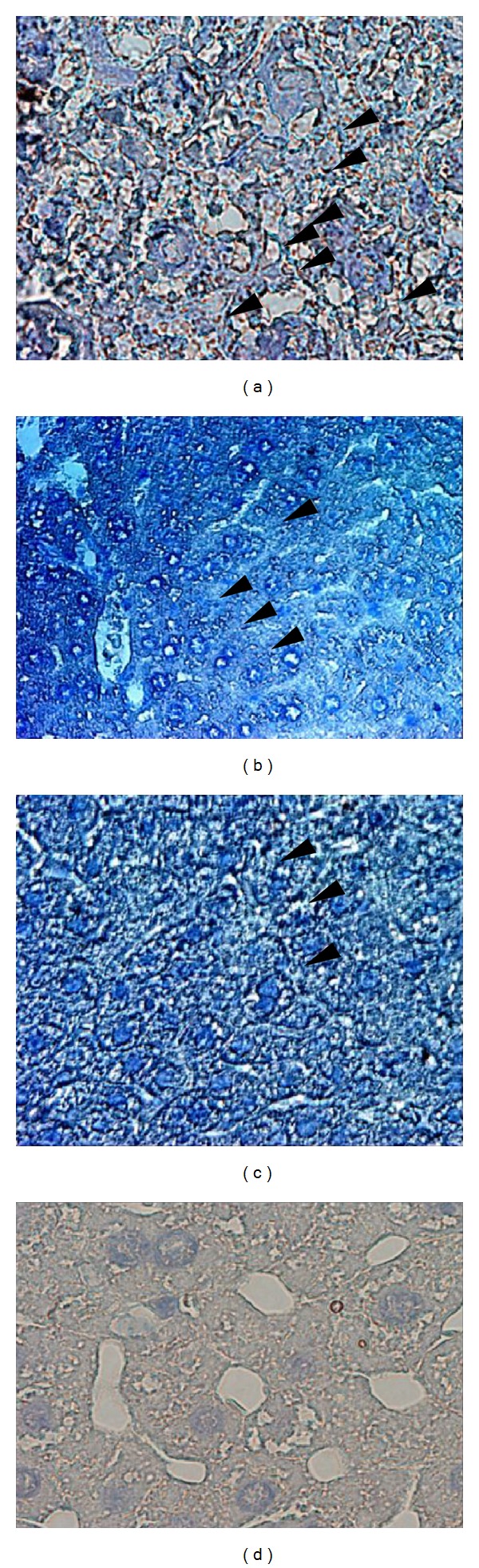
Activity of Ikk *α*/*β* proteins in hepatocytes of C3H mice (a) injected with DEN (×40), (b) Ikk *α*/*β* activity of healthy mice (×40), (c) treated with the decoction subsequent to DEN injection (×40), and (d) negative control; section stained without primary antibody (×40).

As apparent from Figure 5, a significant stimulation of Ikk *α*/*β* activity was observed in mice injected with DEN. This was compatible with the previous studies that demonstrated a stimulation of Ikk *α*/*β* activity by DEN, suggesting that DEN causes NF*κ*B mediated compensatory proliferation of hepatocytes by promoting tumor progression. However, oral treatment of the decoction (1.5 g/kg b.w./day for 4 weeks) significantly inhibited the Ikk *α*/*β* activity in hepatocytes of C3H mice injected with DEN. A marginal activity of Ikk *α*/*β* protein in hepatocytes of healthy mice was observed and it was slightly less than the mice injected with DEN and subsequently treated with the decoction. No reactive foci were observed in tissue sections incubated without primary anti-body, thus confirming the absence of cross reactive sites with the secondary antibody in the tissue sections. 

### 3.7. Effect of the Decoction on Intra-Hepatic Expression of TNF*α*, and IL-6 in Mouse Injected with DEN

**Figure 6:**
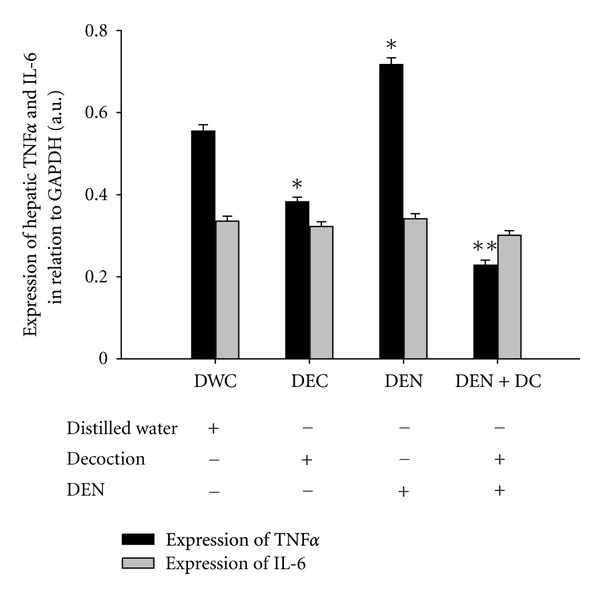
Effect of the decoction on hepatic TNF*α* and IL-6 expression of C3H mice-bearing DEN induced early hepatocarcinogenic changes. Gene expression has quantified using scanning densitometry. Graph illustrates the mRNA expression of TNF*α* and IL-6 of each experimental group relative to expression of GAPDH house-keeping gene. Values are expressed as mean ± SEM (*n* = 8). *P < 0.05 compared to distilled water control (DWC) and **P < 0.05 compared to positive control (DEN) (ANOVA followed by Bonferroni's posttest).

In order to examine the effect of the decoction on immunological liver injury mediated by DEN administration, the expression of TNF*α* and IL-6 was assessed in mouse liver by methods described in Section 2.7.2. As shown in Figure 6, a significant upregulation of intrahepatic TNF*α* expression was observed in C3H mice injected with DEN. It was compatible with previous reports, thus it confirms immunological damage to the liver mediated by DEN. The expression of intra-hepatic TNF*α* in mice previously injected with DEN was significantly attenuated with the oral administration of the decoction (1.5 g/kg b.w./day) for 4 weeks. Intra-hepatic TNF*α* expression was marginally inhibited in mice orally treated only with the decoction (decoction control) in comparison with healthy controls (Figure 6). 

Intra-hepatic IL-6 expression was extremely low in healthy C3H mice. With DEN injection, a moderate surge in intra-hepatic IL-6 expression was noted and was supportive for DEN-mediated immunological liver damage. However, the inhibitory effect of the decoction on intra-hepatic IL-6 expression in C3H mice was insignificant than that observed with respect to TNF*α*. The decoction itself did not cause any significant change in the expression of IL-6 in hepatocytes of healthy C3H mice (Figure 6). Overall expression results of TNF*α* and IL-6 are supportive of previous observation of NF-*κ*B inactivation mediated by the oral decoction treatment.

## 4. Discussion 

A connection between inflammation and cancer has been long suspected [31]. Epidemiological studies have established that many tumors occur in association with chronic infectious diseases, and it is also known that persistent inflammation in the absence of infections increases the risk and accelerates the development of cancer [32]. HCC is a tumor that slowly unfolds on a background of chronic inflammation that is mainly triggered by exposure to infectious agents (hepatotropic viruses) or to toxic compounds (ethanol) [33]. Therefore, it is reasonable to hypothesize that anti-inflammatory effect would be one of the mechanisms by which the decoction comprised of *Nigella sativa *(seeds), * Hemidesmus indicus *(root), and *Smilax glabra *(rhizome) mediates its antihepatocarcinogenic activity.

Carrageenan-induced paw edema is commonly used as a suitable experimental model for evaluating the anti-edematous effect of a test compound [34, 35]. Edema induced by carrageenan is believed to be biphasic [36]; the first phase (1 h) involves the release of serotonin and histamine and the second phase (over 1 h) is linked to neutrophil infiltration and release of products of cyclooxygenase (COX) such as prostaglandins. Continuity between the two phases is provided by kinins [36, 37]. Findings of the present study suggest that the decoction like the reference drug indomethacin exerts prominent inhibition during the second phase of edema. The late phase of edema formation is sensitive to most clinically effective steroids and nonsteroidal anti-inflammatory drugs (NSAIDS) [36]. The decoction used in present study could therefore be therapeutically useful for the treatment of inflammatory conditions. However, it was observed that in comparison to normal controls, a significant reduction in carrageenan induced rat paw edema formation (especially in the early phase) could also be mediated by DEN (hepatocarcinogen) itself. The exact reason for such an inhibition cannot be explained as the extrahepatic inflammatory response during DEN-induced hepatocarcinogenesis has not been well defined. However, a strong intra-hepatic inflammatory response mediated by both type I and II interferons (IFNs) during DEN-induced hepatocarcinogenesis is thought to be responsible for compromised peripheral inflammatory response [38–40]. A further reduction in paw edema formation on oral decoction treatment of rats bearing DEN-induced hepatic damages confirms ability of the decoction to suppress inflammation.

Individual plants of the decoction have previously been reported to inhibit (a) COX and lipoxygenase (5-LO) activities in rat peritoneal leukocytes [41], (b) NO production in LPS-induced murine macrophages [42], (c) *in vivo *and *in vitro *expression of TNF*α* in mice bearing concanavalin A-induced liver damages [43] and in Jurkat cells, respectively [44], and (d) NF-*κ*B activation [45, 46]. However, all these studies used ethanolic extracts of the plant materials and not their aqueous extracts. The present study demonstrates for the first time that an aqueous extract of a polyherbal mixture comprised of the above plants also mediates potent anti-inflammatory activity. 

During inflammation, lysosomal enzymes are released in to the cytosol, causing damage to the surrounding tissues, thereby triggering inflammation. Most of NSAIDS stabilize lysosomal membrane and inhibit the inflammatory process by restricting the release of lysosomal enzymes [47]. The findings of the present study demonstrated that the decoction is capable of stabilizing red-blood cell membrane against hypotonic stress, indicating its ability to prevent rupture or haemolysis of RBCs. Since there is a close similarity of the red-blood cell membrane (RBC) to the lysosomal membrane, protection against hypotonicity or heat-induced lysis of RBC is often extrapolated to stabilization of lysosomal membranes and used as a biochemical index of anti-inflammatory activity [47]. Previous studies have been shown that, flavonoids, triterpenoids, and most other secondary plant metabolites can exhibit analgesic and anti-inflammatory effects as a result of their membrane stabilizing actions [48, 49]. Since the decoction under study has been reported to contain flavonoids, it is not unreasonable to speculate that flavonoids may be one of possible chemical components responsible for the observed membrane stabilizing action. 

An important finding of the present study is the ability of the decoction to inhibit NO production by rat peritoneal cells following exposure to carrageenan. NO is a free radical produced from L-arginine by nitric oxide synthases (NOSs) and plays a central role in inflammation [50]. Uncontrolled release of NO can cause inflammatory destruction of target tissue during an infection [51, 52]. Several compounds such as sesquiterpene lactone from *Artemesia princes *Pampan (Asteraceae), flavin 3,3′-digallate which is a polyphenol from black tea and resveratrol which is a naturally occurring flavanoid from grapes and grapefruits have also been shown to inhibit the inducible nitric oxiside synthase (iNOS) expression [53]. 

Migration or the infiltration of immune cells to the site of inflammation is an important process that takes place in an inflammatory response [54]. In the present study, carrageenan-induced rat peritonitis model [55] was used to assess the *in vivo *inhibitory effects of the decoction on immune cell infiltration. Results clearly indicate a significant inhibitory activity of the decoction on leucocyte migration. 

TNF has a pivotal role in liver pathophysiology because it holds the capacity to induce both hepatocyte death and hepatocyte proliferation, depending on the status of the tissue [56]. Inhibition of TNF expression has been focused on as one of the possible mechanisms to counteract tumor progression [57]. Results of the present study demonstrate a significant inhibition of liver TNF*α* expression in mice that were (a) orally treated with the decoction and (b) injected with DEN and subsequently treated with the decoction. These findings suggest that the inhibition of TNF*α* expression and subsequent events resulting from TNF*α* activation may also be a mechanism by which the decoction mediates its anti-inflammatory action. 

TNF*α* is one of the potent triggers for activating the transcription factor, nuclear factor kappa B (NF-*κ*B) [58]. Upon activation, it upregulates the expression of NF-*κ*B responsive genes [59]. Some of these genes code for cytokines (TNF, IL-1, IL-2, and IL-6), enzymes (inducible nitric oxide synthase, cyclooxygenase 2, and 5-lipoxygenase), and adhesion molecules (Intercellular Adhesion Molecule 1 and Vascular Cell Adhesion Molecule 1) [60] which are closely associated with cell proliferation, angiogenesis, metastasis, tumor promotion, inflammation, and suppression of apoptosis. 

Many carcinogens, inflammatory agents, and tumor promoters have also been shown to activate NF-*κ*B, and resulting tumors demonstrate misregulated NF-*κ*B activity [61]. Therefore, agents capable of suppressing NF-*κ*B activation have been subjected to intense study, anticipating therapeutic promise in tumor management. Results of the present study demonstrate a notable inhibition of hepatic NF-*κ*B activity during chemically induced early hepatocarcinogenesis in C3H mice treated with the decoction. Further, it was observed that the decoction can inhibit the hepatic IKK*β* activity. Therefore, the inhibition of NF-*κ*B by the decoction could be due to inhibition of the TNF-dependent IKK*β* activity. Such an effect would result in inhibition of NF-*κ*B responsive downstream targets. In the present study, this was clearly indicated by the suppression of liver TNF*α* and IL-6 expression in response to oral decoction treatment. However, the effects of decoction on NF-*κ*B activation could have been further supported by the assessment of (a) NF-*κ*B phosphorylation and (b) downstream targets of NF-*κ*B. 

In summary, findings of the present study clearly indicate the presence of potent anti-inflammatory components in the decoction. Anti-inflammatory activity may therefore be one of the mechanisms by which the decoction comprised of *Nigella sativa *(seeds), *Hemidesmus indicus* (root), and *Smilax glabra* (rhizome) mediates its antihepatocarcinogenic activity. 

## Figures and Tables

**Table 1:** Effect of the decoction administration on carrageenan-induced rat paw oedema.

Treatment groups	Increase in paw edema from the baseline (mean ± SEM), (% inhibition)				
	1st h	2nd h	3rd h	4th h	5th h
Control (normal saline)	0.31 ± 0.09	0.43 ± 0.07	0.53 ± 0.09	0.49 ± 0.13	0.41 ± 0.12
Decoction control	0.21 ± 0.04^a∗^ (33%)	0.29 ± 0.08^a∗∗^(30%)	0.36 ± 0.1^a∗∗^ (32%)	0.35 ± 0.12^a∗^ (26%)	0.32±0.12^NS^ (22%)
Diethylnitrosamine control	0.14 ± 0.03 (54%)	0.22 ± 0.06 (48%)	0.26 ± 0.09 (52%)	0.25 ± 0.07 (48%)	0.21 ± 0.09 (49%)
Test (DEN + DC)	0.14 ± 0.01 (60%)	0.21 ± 0.05 (52%)	0.22 ± 0.03^b∗^ (58%)	0.21 ±0.03^b∗^ (57%)	0.19 ± 0.03 (53%)
Indomethacin control	0.17 ± 0.05^a∗∗^ (45%)	0.25 ± 0.07^a∗∗^ (42%)	0.23 ± 0.06^a∗∗^ (57%)	0.22 ± 0.08^a∗∗^ (55%)	0.21 ± 0.04^a∗∗^ (48%)

Decoction was administered for 60 min before subplantar carrageenan injection. The change of footpad volume was determined at 1, 2, 3, 4, and 5 h after irritant injection. Each value represents the mean ± SEM of ten rats per group. Statistically significant difference with respect to the controls and expressed as *P ≤ 0.05, **P ≤ 0.001 (unpaired Student's *t*-test). Figures within parenthesis indicate the differences from normal controls.

^a^Groups 2 and 5 compared with Group 1.

^b^Group 4 compared with Group 3.

**Table 2:** Effect of the decoction on human red cell membrane (HRBC) stability.

	Concentration	% Membrane stability (mean ± SEM)
Decoction	62.5 *μ*g/mL	16.2 ± 1.6
	125 *μ*g/mL	26.4 ± 1.9
	250 *μ*g/mL	39.7 ± 2.8
	500 *μ*g/mL	61.3 ± 4.2
Diclofenac sodium	50 *μ*g/mL	73.9 ± 3.6

Values are expressed as mean ± SEM, *n* = 3/concentration.

**Table 3:** Viability of rat peritoneal cells cultured with different doses of the decoction.

Treatment groups		Number of live cells (%)		
		0 h	1 h	24 h
Control		96.2 ± 1.1	94.6 ± 2.6	89.3 ± 1.2
Decoction	1600 *μ*g/mL	43.6 ± 1.9*	31.7 ± 2.4*	15.4 ± 2.9*
	1200 *μ*g/mL	93.6 ± 1.8	91.5 ± 1.7	87.3 ± 1.3
	600 *μ*g/mL	94.3 ± 2.1	90.8 ± 1.9	88.4 ± 1.6
	300 *μ*g/mL	92.9 ± 2.4	91.1 ± 2.6	87.9 ± 2.1

Values are expressed as mean percentage of living cells ± SEM; *n* = 6/concentration.

*Significant when compared with control; *P* ≤ 0.05.
